# Profiles of exposure to potentially traumatic events in refugees living in Australia

**DOI:** 10.1017/S2045796021000068

**Published:** 2021-02-26

**Authors:** A. Nickerson, Y. Byrow, A. Rasmussen, M. O'Donnell, R. Bryant, S. Murphy, V. Mau, T. McMahon, G. Benson, B. Liddell

**Affiliations:** 1 School of Psychology, University of New South Wales, Sydney, NSW, Australia; 2 Department of Psychology, Fordham University, New York, USA; 3 Phoenix Australia, University of Melbourne, Parkville, VIC, Australia; 4 Australian Red Cross, North Melbourne, VIC, Australia; 5 Settlement Services International, Ashfield, NSW, Australia; 6 Faculty of Medicine, Nursing and Health Sciences, Flinders University, Adelaide, SA, Australia

**Keywords:** Trauma, refugees, torture, posttraumatic stress disorder

## Abstract

**Aims:**

Refugees and asylum-seekers are typically exposed to multiple potentially traumatic events (PTEs) in the context of war, persecution and displacement, which confer elevated risk for psychopathology. There are significant limitations, however, in extant approaches to measuring these experiences in refugees. The current study aimed to identify profiles of PTE exposure, and the associations between these profiles and key demographics, contextual factors (including ongoing stressors, method of travel to Australia and separation from family), mental health and social outcomes, in a large sample of refugees resettled in Australia.

**Methods:**

Participants were 1085 from Arabic, Farsi, Tamil and English-speaking refugee backgrounds who completed an online or pen-and-paper survey in their own language. Constructs measured included PTE exposure, demographics, pre-displacement factors, ongoing stressors, post-traumatic stress disorder symptoms, depression symptoms, anger reactions, plans of suicide and social engagement.

**Results:**

Latent class analysis identified four profiles of PTE exposure, including the torture and pervasive trauma class, the violence exposure class, the deprivation exposure class and the low exposure class. Compared to the low exposure class, participants in the trauma-exposed classes were more likely to be male, highly educated, from Farsi and Tamil-speaking backgrounds, have travelled to Australia by boat, experience more ongoing stressors and report both greater psychological symptoms and social engagement.

**Conclusions:**

This study found evidence for four distinct profiles of PTE exposure in a large sample of resettled refugees, and that these were associated with different demographic, psychological and social characteristics. These findings suggest that person-centred approaches represent an important potential avenue for investigation of PTE exposure in refugees, particularly with respect to identifying subgroups of refugees who may benefit from different types or levels of intervention according to their pre-migration PTE experiences.

Rates of psychological disorders amongst forcibly displaced persons are high compared to host countries' populations (Blackmore *et al*., 2020). Refugees are often exposed to one or more potentially traumatic events (PTEs) in the context of war, persecution or displacement (Johnson and Thompson, 2008; Steel *et al*., 2009), with research highlighting the negative impact of exposure to PTEs on the mental health of refugees and asylum-seekers (Porter and Haslam, 2005; Steel *et al*., 2009). To date, two approaches have generally been employed to measure PTE exposure in refugees. The first involves the use of trauma event checklists, where participants indicate whether they have been exposed to a variety of specific types of trauma events, yielding a count of trauma exposure. Checklist-based methodologies have uncovered a robust relationship between PTE exposure and poor mental health in refugees (Mollica *et al*., 1998; Haldane and Nickerson, 2016; Hecker *et al*., 2018; LeMaster *et al*., 2018). These approaches, however, have critical limitations (Netland, 2001), as they (1) assign equal weighting to each specific type of PTE in the overall trauma exposure score (Houston *et al*., 2011; O'Donnell *et al*., 2017; Rasmussen *et al*., 2018), and (2) measure whether an individual has been exposed to a particular *type* of trauma, without measuring the *frequency* of exposure (Rasmussen *et al*., 2018), making them better equipped to investigate the mental health impact of the *variety* of trauma exposure rather than the *dosage* of trauma exposure (Rasmussen *et al*., 2018).

An alternative approach has focused on investigating the association between types of PTEs and psychological disorders in refugees. Here, researchers have implemented variable-centred data reduction methods (such as factor analysis or principal component analysis) to trauma checklists’ data, identifying groups of PTEs with shared variance, to investigate the association between exposure within these ‘trauma categories’ and mental health. These methods are problematic, however, for several reasons. First, they assume that the population of interest is homogenous in terms of how PTE types influence one another. For example, these approaches would assume that the relationship between PTEs such as torture and exposure to combat is consistent across individuals, whereas these relationships are likely to differ markedly in reality, with some torture survivors having been exposed to combat, while others have not. Furthermore, these approaches assume consistency across a sample in terms of how different types of PTEs impact mental health outcomes (Laursen and Hoff, 2006; O'Donnell *et al*., 2017). Furthermore, the binary (yes/no) nature of checklist data in these studies is incompatible with statistical approaches such as factor analysis and principal component analysis, which, at least in their default modes, assume that data are continuous or ordinal in nature (Rasmussen *et al*., 2018). This may result in inappropriate statistical analyses being applied to the data. Finally, these approaches assume that a hypothesised latent construct is indicated by the variables that are specified to load on this construct. Latent constructs refer to theoretical or unobserved phenomena that cannot be measured directly. Psychological constructs have generally been conceptualised as being latent, whereas objective experiences such as PTEs are by definition not latent (Rasmussen *et al*., 2018).

A useful approach to investigating the co-occurrence of trauma events in refugees, and how profiles of exposure relate to mental health outcomes, is latent class analysis (LCA). LCA is a person-centred statistical approach that, in this context, would allow for the identification of sub-groups of refugees according to their profiles of exposure to PTEs (Collins and Lanza, 2010). LCA has increasingly been seen as the approach of choice in examining the association between trauma and mental health in groups who have been exposed to multiple traumatic events (O'Donnell *et al*., 2017). This approach uses multiple statistical fit indices to determine the optimal number of classes in a particular sample, and facilitates the investigation of predictors and correlates of class membership on a variety of variables of interest (e.g. mental health outcomes). LCA overcomes a number of the limitations in research to date, such that it allows for (1) investigation of the co-occurrence of PTE types without the assumption that there is a latent underlying variable ‘causing’ trauma exposure, (2) determination of the probability of experiencing each PTE type in specific subgroups of refugee participants, instead of yielding a count variable that assumes traumatic events have equal weight in influencing outcomes and (3) facilitating the comparison of models across refugee samples, allowing us to determine whether there are prototypical profiles of PTE exposure that are consistent across groups and contexts.

Although LCA has been used numerous times with other trauma-exposed groups (O'Donnell *et al*., 2017), to date only one study has employed LCA with a refugee sample. Sengoelge and colleagues (2019) identified three classes of PTE exposure in 1215 Syrian refugees resettled in Sweden, namely a ‘multiple violent and non-violent trauma’ class (39.3%), a ‘witnessing violence and multiple non-violent trauma’ class (40.8%) and a ‘low multiple trauma’ class (20.1%), with those in the ‘multiple violent and non-violent trauma’ class showed greatest severity of psychological symptoms. This study was limited, however, by restricted investigation of trauma types, limited consideration of correlates of class membership and a focus on a single refugee group which limits generalisability to other forcibly displaced populations.

Accordingly, an important outstanding question is the extent to which profiles of PTE exposure emerge in a diverse sample of refugees, and how these profiles are related to psychological outcomes. Furthermore, understanding how exposure to particular groups of PTEs impacts social functioning is an important line of enquiry. For example, it has been hypothesised that exposure to interpersonal PTEs, often encountered by refugees (e.g. trauma and sexual assault) leads to greater disruption in ongoing social functioning (Nickerson *et al*., 2014). To date, however, there has been limited evidence to interrogate this hypothesis. Empirical evidence regarding how trauma events co-occur and contribute to both psychological and social outcomes would be important in advancing understanding of the psychosocial impact of the refugee experience, and informing tailored public health programming and interventions for refugees according to their profiles of PTE exposure. This study proposed to examine profiles of PTE exposure in a large sample of refugees from diverse backgrounds residing in Australia, and investigate the association between these profiles and key demographic characteristics, ongoing stressors, psychological and social functioning.

## Method

### Participants

Participants were 1085 individuals living in Australia who (a) were 18+ years, (b) were literate in Arabic, Farsi, Tamil or English (representing >50% of individuals granted refugee status in Australia between 2012 and 2015 (DIBP, 2014)), (c) arrived in Australia since January 2011 and (d) were from a refugee or asylum-seeking background. We recruited participants through advertisements at refugee support services across Australia, social media platforms (i.e. Facebook) and snowball sampling, which has been found to be effective for difficult-to-access populations (Sadler *et al*., 2010). Participants in this study had entered Australia via two pathways (Nickerson *et al*., 2019). Approximately three-quarters of the sample (*n* = 826, 76.1%) had applied for and been granted refugee status prior to arriving in Australia (via the United Nations High Commissioner for Refugees or Australia's humanitarian programme). These individuals had generally been provided with permanent visas, allowing them to remain in Australia indefinitely. The second pathway involved arriving in Australia without a valid visa (often via boat), or holding a non-refugee visa and subsequently applying for refugee status (*n* = 260, 23.9%). These individuals often face lengthy processing periods and may only be granted temporary visas, which afford restricted access to services and support.

### Measures

Gold-standard translation and blind back-translation methods were used (World Health Organization, n.d.). Translated measures were pilot-tested with at least two individuals with varying education levels from each language group. These individuals had previously taken part in research studies undertaken by the research group, and had expressed interest in being involved in future research. This process resulted in only minor changes, mostly relating to ensuring the complexity of language used in specific items was appropriate for participants with a wide range of educational attainments.

*Demographics* were collected on participants' age, gender, time in Australia, method of arrival (boat or airplane), language, marital status, highest level of education attained and family separation.

*Exposure to PTEs* was measured using the 16-item Harvard Trauma Questionnaire (Mollica *et al*., 1992). Participants indicated whether they had experienced, witnessed or learned about traumatic events commonly experienced by refugees. In this study, we defined PTE exposure as having experienced the event directly. Each PTE type was scored dichotomously (i.e. 0 = *did not experience*; 1 = *experienced*).

*Ongoing stressors* were measured using a 36-item version of the Post-Migration Living Difficulties Checklist adapted for the Australian context (Silove *et al*., 1998; Steel *et al*., 1999). Participants rated each item on a scale on a 5-point scale (1 = *was not a problem*/*did not happen*, *5* = *a very serious problem*). A stressor was considered to be present if rated as at least 3 (*a moderately serious problem*), consistent with past approaches using this scale (Tay *et al*., 2019). A count of ongoing stressors was computed, ranging from 1 to 36.

*Posttraumatic stress symptoms* were measured using the 16-item Posttraumatic Diagnostic Scale (Foa, 1996). As the DSM-5 version of the PDS was not available when the study commenced, four items were added to the PDS to measure post-traumatic stress disorder (PTSD) symptoms new to DSM-5. Participants indicated on a 4-point scale how often each symptom bothered them in the past month (from 0 = *not at all or only once*, to 3 = *5 or more times a week*/*almost always*). A sum score of PTSD symptoms was calculated, ranging from 0 to 60 (*α* = 0.96).

*Depression symptoms* were measured using the 9-item Patient Health Questionnaire (Kroenke *et al*., 2001) Participants indicated on a 4-point scale (0 = *not at all*, 3 = *nearly every day*) how often they had been bothered by symptoms in the past 2 weeks. A sum score of depression symptoms was calculated, ranging from 0 to 27 (*α* = 0.93).

*Plans of suicide* were measured using a single item to which participants responded yes/no: ‘*in the past two weeks, have you made specific plans for suicide, intended to take your own life, or taken steps towards putting your plan to kill yourself into action*’. Participants who responded yes were contacted by telephone by a clinical psychologist and referred to appropriate support.

*Feelings of anger* were measured using the five-item Dimensions of Anger Reactions (DAR-5) scale (Forbes *et al*., 2014). Participants indicated on a 5-point scale how often they experienced each of the items (1 = *none or almost none of the time*, 5 = *all or almost all of the time*). This scale ranged from 5 to 25. A mean score for feelings of anger was calculated (*α* = 0.99).

*Disability* was measured using the 12-item World Health Organization Disability Assessment Schedule 2.0 (WHODAS 2.0) (World Health Organization, 2000). Participants indicated how much difficulty they had doing daily activities over the past 30 days on a 5-point scale (0 = *none*, 5 = *extreme or cannot do*). Items were summed to create a total disability score, ranging from 0 to 60 (*α* = 0.91).

*Social engagement* was measured using the 10-item Short Social Capital Assessment Tool (SASCAT) (Harpham *et al*., 2002; De Silva *et al*., 2007). This scale measures the number of types of (1) group participants were active members of, (2) groups from which participants received emotional or economic assistance and (3) individuals from which participants received emotional or economic assistance in the past 12 months. A total count of the number of groups or individuals participants engaged within the past 12 months was created, ranging from 0 to 33.

### Procedure

Data collection was undertaken between April 2015 and January 2018. Measures were administered online via the KeySurvey platform, and took 45 min to 1 h to complete. For participants who did not have access to the internet, hard copies of the informed consent documents and survey were posted. Participants received an $AUD25 shopping voucher to compensate for costs associated with undertaking the survey. All procedures were approved by the UNSW Human Research Ethics Committee, HC14106. The authors assert that all procedures contributing to this study comply with the ethical standards of the relevant national and institutional committees on human experimentation and with the Helsinki Declaration of 1975, as revised in 2013.

### Data analysis

LCA was used to identify profiles of exposure to 16 types of PTEs amongst refugee participants using Mplus version 8.2 (Muthen and Muthen, 1998–2019). Mplus uses a full information maximum likelihood estimator with robust standard errors to account for missing data. The most parsimonious (one-class) solution is first fit, followed by successive models with increasing number of classes. Model fit was evaluated using (1) lower values on the Akaike information criteria (AIC), the Bayesian information criteria (BIC) and the sample size-adjusted Bayesian information criteria (SS-BIC), (2) higher entropy values, (3) non-significant Lo–Mendell–Rubin likelihood ratio test (LMR-LRT) and Vuong–Lo–Mendell–Rubin likelihood ratio test (V-LRT) and (4) parsimony and interpretability. The following values were used to evaluate probabilities of endorsing exposure to specific PTEs: ⩾0.60: high probability of endorsement; ⩽0.59 and ⩾0.16: moderate probability of endorsement and ⩽0.15: low probability of endorsement. After determining the optimal class solution, multinomial logistic regression was used to investigate predictors (via the R3Step method) and distal outcomes (using the ‘Bolck, Croon and Hagenaars’ (BCH) approach) associated with class membership in the AUXILIARY command in Mplus (Asparouhov and Muthen, 2014).

## Results

### Participant characteristics and PTE exposure

Participant characteristics are presented in Table 1. The most commonly-experienced PTEs (see Table 2) were lack of food or water (*n* = 454, 44.2%) and being close to death (*n* = 441, 43.0%). Substantial numbers of participants had also been exposed to torture (*n* = 228, 22.2%).

**Table 1.** Participant characteristics

Demographics	*N*	%
Age	Mean = 38.11	s.d. = 11.79
Gender (female)	465	42.9
Language		
Arabic	741	68.3
Farsi	186	17.1
English	104	9.6
Tamil	54	5.0
Country of birth		
Iraq	589	54.3
Iran	174	16.0
Syria	162	14.9
Sri Lanka	58	5.3
Afghanistan	37	3.4
Other	65	6.0
No immediate family in Australia	251	23.1
Marital status		
Married/in a relationship	773	71.2
Not married/in a relationship	311	27.7
Education		
Little/no formal education	48	4.5
Primary school	122	11.4
High school	378	35.3
University	428	39.9
Other training (vocational, apprenticeship)	96	8.8
Employment		
Employed	480	44.2
Unemployed	188	17.3
Studying	418	68.5
Time in Australia	*M* = 1.98	s.d. = 1.65
Visa status		
Secure (e.g. Permanent Residency, Australian Citizenship)	826	76.1
Insecure (i.e. temporary visa, bridging visa, no visa)	260	23.9

**Table 2.** Potentially traumatic events

	*N*	%
Lack of food or water	454	44.2
Being close to death	441	43.0
Ill health without access to medical care	403	39.3
Lack of shelter	388	37.8
Serious injury	325	31.7
Forced separation from family members	299	29.1
Combat situation	293	28.6
Lost or kidnapped	252	24.6
Forced isolation from others	247	24.1
Imprisonment	244	23.8
Torture	228	22.2
Unnatural death of family or friend	205	20.0
Brain washing	162	15.8
Murder of family or friend	161	15.7
Murder of stranger or strangers	140	13.6
Rape or sexual abuse	127	12.4

### Latent class analysis

#### Unconditional models

A four-class solution best fits the data (Table 3). AIC, the BIC and the SS-BIC showed substantial reductions until the four-class solution, and smaller reductions or increases thereafter. Entropy was relatively higher in the four-class compared to the three and five-class solutions. The V-LRT and LMR-LRT indicated that the four-class solution showed significantly better fit than the five-class solution, but there were no significant differences in fit between subsequent solutions.

**Table 3.** Fit statistics for LCA

	AIC	BIC	SS-BIC	Entropy	V-LRT	LMR-LRT
1 class	16 082.907	16 162.7	16 111.8			
2 class	12 929.65	13 094.1	12 989.3	0.907	<0.001	<0.001
3 class	12 496.099	12 745.3	12 586.5	0.839	<0.001	<0.001
4 class	12 340.401	12 674.4	12 461.6	0.848	0.0008	0.0008
5 class	12 221.544	12 640.3	12 373.5	0.841	0.2925	0.2953
6 class	12 164.749	12 668.2	12 347.4	0.843	0.095	0.0964
7 class	12 122.989	12 711.2	12 336.4	0.826	0.1715	0.1738

**Fig. 1.**
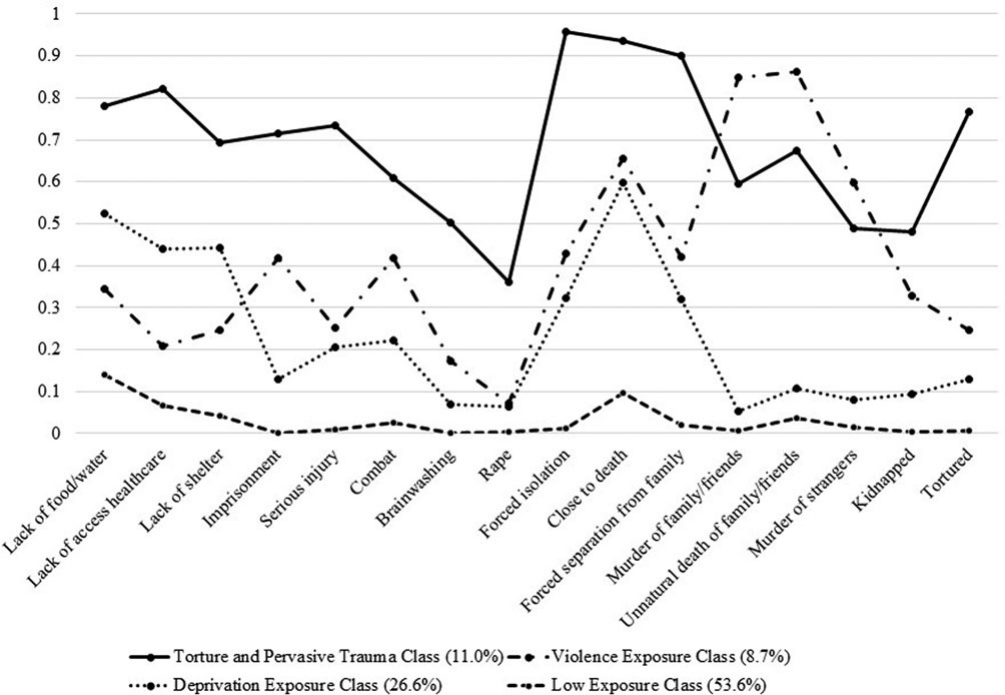
Four-class solution for LCA.

Conditional probabilities for PTE exposure according to class membership are presented in Fig. 1 (see Appendix Table A). Participants in the torture and pervasive trauma exposure class (TPT class; 11.0%) reported a high probability of being exposed to all trauma types (including torture), and a moderate probability of being exposed to brain-washing, rape, murder of strangers and kidnapping.

Participants in the violence exposure class (VE class; 8.7%) had a high probability of being close to death and experiencing the murder of others, and a moderate probability of being exposed to all other trauma types, with the exception of rape.

Participants in the deprivation exposure class (DE class; 26.6%) had a high probability of being close to death, and a moderate probability of being exposed to all other types of traumatic events. Notably, this group had a relatively higher probability of being exposed to lack of food or water, ill health without access to medical care and lack of shelter, compared to other trauma types.

Participants in the low trauma exposure class (LE class; 53.6%) had a low probability of being exposed to all trauma types, and a moderate probability of being exposed to lack of food and water.

### Predictors of class membership

Compared to the LE class, the TPT class were more likely to be male, speak Arabic, Farsi or Tamil compared to English, be married and have completed tertiary education (Table 4). Compared to the DE class, the TPT class were more likely to be male, and speak Tamil or Farsi. Compared to the VE class, the TPT class were more likely to speak Tamil and be married. Compared to the LE class, the VE class were more likely to be male, less likely to speak Arabic and more likely to speak Farsi than English, and more likely to have completed tertiary education. Compared to the LE class, the DE class were more likely to be male, less likely to speak Arabic than English, and more likely to have completed tertiary and/or other education.

**Table 4.** Predictors of class membership

	Torture and pervasive trauma				Violence exposure				Deprivation exposure			
	Est (s.e.)	*p*	OR	95% CI	Est (s.e.)	*p*	OR	95% CI	Est	*p*	OR	95% CI
*Vs Low exposure class*												
Age	0.02 (0.01)	0.12	1.22	1.19–1.25	−0.01 (0.01)	0.908	1.00	0.97–1.02	0.01 (0.01)	0.493	1.01	0.99–1.03
Sex	−1.27 (0.29)	<0.001	0.28	0.16–0.50	−0.87 (0.30)	0.003	0.42	0.23–0.75	−0.57 (0.20)	0.004	0.56	0.38–0.83
*Language*												
Arabic	−2.03 (0.46)	<0.001	0.13	0.05–0.33	−1.45 (0.42)	<0.001	0.24	0.10–0.53	−1.29 (0.4)	<0.001	0.28	0.14–0.54
Tamil	1.48 (0.45)	0.001	4.4	1.81–10.71	0.43 (0.47)	0.354	1.54	0.61–3.87	0.06 (0.41)	0.893	1.06	0.47–2.38
Farsi	2.38 (0.69)	0.001	10.77	2.81–41.33	1.52 (0.77)	0.047	4.59	1.02–20.66	0.76 (0.74)	0.303	2.13	0.50–9.01
Married	1.05 (0.31)	0.001	2.87	1.55–5.29	0.11 (0.32)	0.728	1.12	0.60–2.08	0.16 (0.24)	0.491	1.17	0.74–1.86
*Education*												
Primary	1.31 (0.86)	0.124	3.72	0.70–19.88	0.67 (0.66)	0.312	1.95	0.53–7.15	0.14 (0.76)	0.851	1.15	0.26–5.13
Secondary	1.30 (0.75)	0.083	3.67	0.84–15.97	0.03 (0.62)	0.964	1.02	0.31–3.45	1.02 (0.66)	0.121	2.76	0.77–9.98
Tertiary	1.90 (0.76)	0.012	6.67	1.51–29.45	1.11 (0.55)	0.043	3.04	1.03–8.95	1.30 (0.66)	0.048	3.68	1.02–13.35
Other	2.35 (0.84)	0.005	10.43	2.02–53.92	0.35 (0.79)	0.655	1.42	0.30–6.68	1.76 (0.71)	0.013	5.84	1.44–23.56
*Vs Deprivation exposure class*												
Age	0.01 (0.01)	0.308	1.01	0.99–1.04	−0.01 (0.02)	0.595	0.99	0.96–1.02				
Sex	−0.70 (0.31)	0.022	0.50	0.27–0.90	−0.30 (0.33)	0.356	0.74	0.39–1.40				
*Language*												
Arabic	−0.75 (0.45)	0.096	0.47	0.20–1.14	−0.16 (0.42)	0.704	0.85	0.38–1.94				
Tamil	1.43 (0.43)	0.001	4.16	1.78–9.73	0.38 (0.47)	0.423	1.46	0.58–3.70				
Farsi	1.62 (0.56)	0.004	5.05	0.69–15.11	0.77 (0.71)	0.283	2.15	0.53–8.70				
Married	0.89 (0.32)	0.005	2.44	1.30–4.56	−0.05 (0.365)	0.881	0.95	0.48–1.87				
*Education*												
Primary	1.17 (0.98)	0.233	3.23	0.47–22.06	0.53 (0.91)	0.564	1.69	0.28–10.09				
Secondary	0.28 (0.83)	0.732	1.32	0.26–6.79	−0.99 (0.81)	0.222	0.37	0.08–1.82				
Tertiary	0.60 (0.84)	0.479	1.81	0.35–9.41	−0.19 (0.76)	0.801	0.83	0.19–3.64				
Other	0.58 (0.89)	0.515	1.79	0.31–10.25	−1.41 (0.95)	0.137	0.24	0.04–1.56				
*Vs Violence exposure class*												
Age	0.02 (0.02)	0.217	1.02	0.99–1.06								
Sex	−0.40 (0.39)	0.301	0.67	0.31–1.43								
*Language*												
Arabic	−0.59 (0.55)	0.284	0.56	0.19–1.63								
Tamil	1.05 (0.53)	0.049	2.85	1.01–8.06								
Farsi	0.85 (0.69)	0.216	2.35	0.61–9.08								
Married	0.94 (0.39)	0.017	2.57	1.19–5.56								
*Education*												
Primary	0.65 (1.00)	0.52	1.91	0.27–13.61								
Secondary	1.27 (0.93)	0.169	3.57	0.58–21.89								
Tertiary	0.79 (0.89)	0.375	2.19	0.39–12.42								
Other	1.99 (1.09)	0.067	7.33	0.87–62.08								

### Distal outcomes associated with class membership

Compared to the LE class, the TPT class were more likely to have travelled by boat, be separated from family, exposed to more types of PTEs and report more ongoing stressors, more severe psychological symptoms and greater social capital (Table 5). Compared to the VE class, the TPT class were more likely to have travelled by boat, be separated from their immediate family, be exposed to more PTEs, and report greater ongoing stressors, and more severe PTSD symptoms, depression symptoms, anger and disability symptoms. Compared to the DE class, the TPT class were more likely to have travelled by boat, be separated from all of their immediate family, be exposed to more PTEs and report more ongoing stressors, more severe psychological symptoms and greater social capital.

**Table 5.** Experiences and outcomes associated with class membership

	TPT class *M* (s.e.)	Low Exp class *M* (s.e.)	Chi square	*p* value	Viol Exp class *M* (s.e.)	Low Exp class *M* (s.e.)	Chi square	*p* value	Depriv class *M* (s.e.)	Low Exp class *M* (s.e.)	Chi square	*p* value
PTE exposure	11.43 (0.11)	0.23 (0.04)	2543.90	<0.001	6.13 (0.24)	0.23 (0.04)	587.42	<0.001	4.01 (0.11)	0.23 (0.04)	969.74	<0.001
Method of travel (boat)	1.68 (0.05)	1.08 (0.01)	156.51	<0.001	1.39 (0.06)	1.08 (0.01)	28.53	<0.001	1.27 (0.03)	1.08 (0.01)	29.28	<0.001
Family separation	0.41 (0.05)	0.88 (0.02)	88.79	<0.001	0.65 (0.06)	0.88 (0.02)	16.02	<0.001	0.73 (0.03)	0.88 (0.02)	18.55	<0.001
Ongoing stressors	17.49 (0.93)	4.42 (0.31)	177.32	<0.001	11.93 (0.98)	4.42 (0.31)	54.12	<0.001	11.14 (0.63)	4.42 (0.31)	82.41	<0.001
PTSD	35.11 (1.71)	7.31 (0.51)	244.16	<0.001	22.44 (1.80)	7.31 (0.51)	65.44	<0.001	20.82 (1.06)	7.31 (0.51)	116.65	<0.001
Depression	1.72 (0.08)	0.59 (0.03)	192.32	<0.001	1.32 (0.09)	0.59 (0.03)	59.37	<0.001	1.15 (0.05)	0.59 (0.03)	80.52	<0.001
Anger	11.91 (0.45)	7.39 (0.14)	93.89	<0.001	10.12 (0.49)	7.39 (0.14)	29.2	<0.001	9.58 (0.27)	7.39 (0.14)	46.38	<0.001
Disability	19.68 (1.16)	12.10 (0.45)	37.38	<0.001	16.07 (1.09)	12.10 (0.45)	11.3	0.001	17.18 (0.68)	12.10 (0.45)	34.32	<0.001
Suicidality	0.17 (0.04)	0.01 (0.01)	21.4	<0.001	0.09 (0.03)	0.01 (0.01)	7.23	0.007	0.02 (0.01)	0.01 (0.01)	3.3	0.069
Social capital	3.32 (0.37)	1.41 (0.10)	24.77	<0.001	2.55 (0.35)	1.41 (0.10)	9.94	0.002	2.30 (0.18)	1.41 (0.10)	17.35	<0.001
	TPT class M (s.e.)	Depriv class M (s.e.)	Chi square	*p* value	Viol Exp class M (s.e.)	Depriv class M (s.e.)	Chi square	*p* value				
PTE exposure	11.43 (0.11)	4.01 (0.11)	895.9	<0.001	6.13 (0.24)	4.01 (0.11)	587.42	<0.001				
Method of travel (boat)	1.68 (0.05)	1.27 (0.03)	52.6	<0.001	1.39 (0.06)	1.27 (0.03)	3.23	0.072				
Family separation	0.41 (0.05)	0.73 (0.03)	29.80	<0.001	0.65 (0.06)	0.73 (0.03)	1.28	0.259				
Ongoing stressors	17.49 (0.93)	11.14 (0.63)	30.71	<0.001	11.93 (0.98)	11.14 (0.63)	0.44	0.509				
PTSD	35.11 (1.71)	20.82 (1.06)	48.77	<0.001	22.44 (1.80)	20.82 (1.06)	0.56	0.454				
Depression	1.72 (0.08)	1.15 (0.05)	36.45	<0.001	1.32 (0.09)	1.15 (0.05)	2.59	0.108				
Anger	11.91 (0.45)	9.58 (0.27)	19.49	<0.001	10.12 (0.49)	9.58 (0.27)	0.89	0.345				
Disability	19.68 (1.16)	17.18 (0.68)	3.34	0.067	16.07 (1.09)	17.18 (0.68)	0.70	0.404				
Suicidality	0.17 (0.04)	0.02 (0.01)	14.77	<0.001	0.09 (0.03)	0.02 (0.01)	3.73	0.053				
Social capital	3.32 (0.37)	2.30 (0.18)	5.97	0.014	2.55 (0.35)	2.30 (0.18)	0.39	0.533				
	TPT class *M* (s.e.)	Viol Exp class *M* (s.e.)	Chi square	*p* value								
PTE exposure	11.43 (0.11)	6.13 (0.24)	253.23	<0.001								
Method of travel (boat)	1.68 (0.05)	1.39 (0.06)	14.66	<0.001								
Family separation	0.41 (0.05)	0.65 (0.06)	10.18	0.001								
Ongoing stressors	17.49 (0.93)	11.93 (0.98)	15.84	<0.001								
PTSD	35.11 (1.71)	22.44 (1.80)	24.44	<0.001								
Depression	1.72 (0.08)	1.32 (0.09)	10.26	<0.001								
Anger	11.91 (0.45)	10.12 (0.49)	6.93	0.01								
Disability	19.68 (1.16)	16.07 (1.09)	4.82	0.03								
Suicidality	0.17 (0.04)	0.09 (0.03)	2.37	0.12								
Social capital	3.32 (0.37)	2.55 (0.35)	2.15	0.14								

Compared to the LE class, the VE class were more likely to have travelled boat, be separated from all of their immediate family, be exposed to more PTEs, report more ongoing stressors, more severe psychological symptoms and report greater social capital. The VE class had been exposed to more types of PTEs than those in the DE class.

Compared to the LE class, the DE class were more likely to have travelled by boat, be separated from all of their immediate family, be exposed to more PTEs, report more ongoing stressors, more severe psychological symptoms and report greater social capital.

## Discussion

To our knowledge, this is the first study to investigate profiles of PTE exposure amongst a large and diverse sample of resettled refugees. Findings indicated that qualitatively different classes of PTE exposure emerged from the data, and that these classes were associated with distinctive characteristics, experiences and mental health outcomes. These findings highlight the heterogeneous nature of PTEs experienced by the current sample. A key finding from this study is that PTE profiles were strongly tied to personal characteristics and contextual factors, suggesting that there are particular groups who are especially vulnerable to polytrauma exposure. The finding that those in the trauma-exposed classes were more likely to be male and have higher education may represent the greater involvement of men with higher education in political activities, trauma in the context of employment and combat settings. Furthermore, the finding that individuals in the TPT class were more likely to be from Farsi or Tamil-speaking backgrounds likely reflects the prevalence of torture in these settings as a way of suppressing political dissidence (Hamid, 2017; Wintour, 2018). Individuals in each of the trauma-exposed classes were also more likely to have travelled to Australia by boat, likely reflecting high exposure to trauma during the journey (e.g. witnessing drownings at sea, and lack of food and water (Hauff and Vaglum, 1993; Nickerson *et al*., 2019)). In addition, Australian immigration policy dictated that all refugees who travelled by boat to Australia without a valid visa during the data collection phase of the study were subsequently held in immigration detention or offshore processing centres (Parliament of Australia, 2013–2014), with this experience being associated with exposure to adverse events such as witnessing self-harm and physical assault, discrimination and lack of access to medical care (Steel *et al*., 2006). These findings extend on past research that has documented greater PTE exposure in refugees with insecure visas, many of whom had arrived in Australia by boat (Steel *et al*., 2006; Nickerson *et al*., 2019), to suggest that these individuals also experience a greater diversity of PTE exposure. Membership in the trauma-exposed classes was also associated with greater ongoing stressors in the post-displacement environment. This is consistent with theoretical models and empirical evidence suggesting that trauma exposure in settings of persecution and conflict are linked to greater stressors in the post-conflict and post-migration environment (Miller and Rasmussen, 2010, 2017). These findings suggest a heightened burden for trauma-exposed individuals who must cope with the psychological effects of extensive prior trauma exposure, but also managing a myriad of stressors in the post-migration environment.

It is notable that the TPT class exhibited the poorest mental health outcomes in this study, with significantly higher PTSD symptoms, depression symptoms, anger and disability than all other classes, and greater plans of suicide than the deprivation and low trauma exposure classes. This finding is consistent with research demonstrating that greater diversity of trauma experiences is associated with greater psychological distress (Porter and Haslam, 2005; Steel *et al*., 2009; Chung *et al*., 2018), and research linking interpersonal trauma and suicidality in non-refugee trauma-affected samples (LeBouthillier *et al*., 2015; Beristianos *et al*., 2016; Asgeirsdottir *et al*., 2018). It is also notable that the DE class showed poorer mental health outcomes than the low exposure class. This is consistent with other research indicating that exposure to non-interpersonal adverse events such as lack of food, water, shelter and medical care can have significant negative mental health effects for refugees and other trauma-affected groups (Forbes *et al*., 2012*a*, *b*; Haldane and Nickerson, 2016). These findings suggest that it is critically important to make mental health services available for refugees with heterogeneous trauma experiences.

An important finding in this study was that those in the trauma-exposed classes reported greater social capital, suggesting that they were proactive in engaging with social groups both within and external to their communities. This finding is in contrast to past research suggesting that there is an inverse relationship between PTSD symptoms and social capital among trauma survivors (Wind and Komproe, 2012; Flores *et al*., 2014; Gao *et al*., 2018). There are several potential explanations for this finding. First, it is likely the case that individuals in these classes (who were also more likely to be separated from their families) required more practical and emotional support than those in the low exposure class as a function of their psychological symptoms, which may have led them to connect with other individuals and groups as a way of addressing these needs. This is consistent with research suggesting that greater symptoms of posttraumatic stress are associated with greater help-seeking among trauma survivors (Guina *et al*., 2018; Takaoka *et al*., 2018). Second, our finding that individuals in the trauma-exposed classes showed higher levels of education suggests that there may be a link between education level, trauma exposure and social functioning. For example, it may be the case that individuals who are in leadership positions in their cultural, religious or political groups are more likely to be targeted for persecution. These individuals may, in turn, have a stronger proclivity to building social connections in the post-migration environment, potentially accounting for the greater group membership. Overall, this finding challenges the conception that mental health problems arising from trauma exposure necessarily lead to social impairment, highlighting the potential positive social and cultural contributions trauma-exposed refugees can make in their countries of resettlement (RCOA, 2010). It is important to note, however, that it is not possible to rule out a methodological explanation for this finding. Specifically, the use of snowball sampling methods, while effective at engaging difficult-to-access populations may have led to individuals being recruited into the study who had greater levels of social connection. This may have influenced the study findings relating to social capital, and it is important that further research implementing representative or random sampling methods be undertaken to investigate the association between PTE exposure and social capital.

From a methodological standpoint, the current study highlights the potential for latent class approaches to increase our understanding of the diversity of PTEs experienced by refugees. Focusing exclusively on the number of types of PTEs to which an individual has been exposed, as has been done in a number of past studies, would have failed to detect the qualitatively distinct profiles of PTE exposure, and precluded nuanced investigation of the typical characteristics of individuals who had experienced these events, nor their specific mental health consequences. Although this study was limited in its extent to comprehensively investigate trauma types and to index frequency of PTE exposure, this represents an important step in overcoming limitations to trauma measurement in refugee groups to date, and enhance our knowledge regarding the nature of the refugee experience. Future research could combine an investigation of type and frequency of exposure to PTEs to gain a comprehensive understanding of how these two factors interact to influence mental health. Furthermore, it is important to note that, in situations of ongoing war, systemic violence and/or human rights violations, exposure to PTEs may be repeated and occur over an extended period of time. Furthermore, social structures that may previously have been considered protective or benevolent (e.g. schools, hospitals and places of workshop) may become contexts of exposure to PTEs or other types of adversity (Nickerson *et al*., 2014). The pervasiveness of PTE exposure in these areas where persecution and conflict are ongoing may thus fundamentally affect personal and communal resources, and the specific strategies enacted by individuals and communities to cope with ongoing war and persecution. Measuring the impact of these experiences represents a significant challenge for the field, and is likely to be much more complex than considering simple indices of type and frequency of PTE exposure. Nevertheless, understanding how broad and pervasive PTE exposure impacts on individuals' and communities' wellbeing and capacity to goal is an important goal, with great potential for informing multi-level interventions in persecution and conflict-related contexts.

The current study had several limitations. Although the refugee groups in this study were broadly representative, there are considerations relating to both the homogeneity and heterogeneity of the sample in terms of generalisability. First, the sample was limited to four language groups from two global regions, and literacy was an inclusion criterion for the study, meaning that the experiences of refugees with lower education and literacy levels were not captured. In terms of heterogeneity, this sample comprised of both individuals who had entered Australia via offshore and onshore pathways, resulting in a considerable variability in the sample in terms of country of origin, travel to Australia, migration process and available support. The heterogeneous nature of the sample may thus limit specificity of findings. In addition, the snowball sampling method used in this study, although effective at accessing ‘hidden’ populations (Sadler *et al*., 2010), may have resulted influenced the results in this study. For example, it may be the case that this sampling method may have led to individuals with higher levels of education and/or social connectedness taking part, potentially accounting for the higher level of social capital in the greater trauma-exposed classes. Future research implementing random or representative sampling methods should be undertaken to replicate these results.

Upon reflection we noted that several PTEs on the HTQ do not described specific types of events (e.g. ‘Torture’ and ‘Being close to death’), and that it was possible that one event might be described by more than one item (e.g. ‘Unnatural death of family or friend’ and ‘Murder of family or friend’; ‘Torture’ and ‘Brain washing’). It may have been the case that some association observed in the LCA was due to such nonspecific and overlapping item content, and not the co-occurrence of events within group profiles *per se*. In addition, we did not specifically measure exposure to PTEs in the post-migration environment. It may have been the case, for example, that specific differential profiles of PTE exposure emerged when considering PTE exposure prior to and after arrival in Australia separately. In addition, it is possible that PTE exposure prior to arrival in Australia may have predicted different PTE exposure in the post-migration environment, and that PTE exposure in the post-migration environment may mediate the association between pre-migration PTE exposure and psychological outcomes. Further research should consider differential PTE exposure at specific phases of the migration process to examine this question. There is also evidence that rates of psychopathology increase in the years following arrival in the host country, and that there is an association between psychological distress and poorer social and economic outcomes (WHO, 2018). Thus, investigation of the association between PTE profiles, ongoing stressors and psychopathology over time following resettlement is an important area for future enquiry. Furthermore, the measure of plans of suicide in this study was limited to a single dichotomous item. Future research should implement more comprehensive and validated tools to assess the construct of suicidality. Another limitation of this study relates to the mental health outcomes included in the analysis. Although we attempted to include a comprehensive set of psychological constructs (comprising PTSD, depression, anger and plans of suicide), these outcomes were indexed by western-derived measures, and did not index culturally-specific symptoms or idioms of distress, nor social or functional outcomes. Given the critique of employing diagnostic categories developed on the basis of research with western populations with war-affected groups from non-western countries (in particular, PTSD which has been argued to pathologise normative stress responses and divert the focus from systemic violence and injustice to the level of the individual (Bracken *et al*., 1995; Summerfield, 1999; Loughry and Eyber, 2003)), it is important that future research considers the association between PTE profiles and a broader range of ecologically valid psychological, social and functional outcomes.

This study found evidence for four distinct profiles of PTE exposure in a large sample of resettled refugees. Although further research is required to determine whether similar profiles emerge in other displaced and war-affected populations, this study provides initial evidence that these profiles differed in terms of both *type* and *variety* of PTE exposure, and were associated with different demographic, psychological and social characteristics. These findings have important potential implications for both future research directions and clinical approaches. First, the results underscore the potential utility of person-centred approaches in investigating PTE exposure in refugees, with these methods allowing for a more nuanced understanding of how variety and type of PTE experiences interact, and relate to key psychological and social outcomes. Second, the identification of specific PTE profiles and associated characteristics provide a potential framework for identifying subgroups of refugees who may benefit from different types or levels of interventions according to their pre-migration experiences. Overall, this study underscores the complexity of the refugee experience, and how exposure to many different types of traumatic events can shape the psychological and social experience of individuals fleeing from persecution and war.

## Data Availability

Data are available from the first author at reasonable request.

## Appendix

**Table A1.** Conditional Probabilities and Standard Errors for Class Membership

	Torture and Pervasive Trauma Class (11.0%)	Violence Exposure Class (8.7%)	Deprivation Class (26.6%)	Low Exposure Class (53.6%)
Lack of food/water	0.78 (0.05)	0.35 (0.07)	0.52 (0.04)	0.14 (0.03)
Lack of access healthcare	0.82 (0.05)	0.21 (0.06)	0.44 (0.04)	0.07 (0.02)
Lack of shelter	0.69 (0.06)	0.25 (0.07)	0.44 (0.04)	0.04 (0.01)
Imprisonment	0.72 (0.05)	0.42 (0.07)	0.13 (0.03)	0.00 (0.01)
Serious injury	0.73 (0.05)	0.25 (0.06)	0.21 (0.04)	0.01 (0.01)
Combat	0.61 (0.05)	0.42 (0.07)	0.22 (0.03)	0.03 (0.01)
Brainwashing	0.50 (0.05)	0.17 (0.05)	0.07 (0.02)	0.00 (0.01)
Rape	0.36 (0.05)	0.07 (0.04)	0.06 (0.02)	0.00 (0.01)
Forced isolation	0.96 (0.02)	0.43 (0.07)	0.32 (0.05)	0.01 (0.01)
Close to death	0.94 (0.03)	0.66 (0.06)	0.60 (0.05)	0.10 (0.02)
Forced separation from family	0.90 (0.03)	0.42 (0.08)	0.32 (0.05)	0.02 (0.01)
Murder of family/friends	0.6 (0.06)	0.85 (0.06)	0.05 (0.03)	0.01 (0.01)
Unnatural death of family/friends	0.67 (0.06)	0.86 (0.06)	0.11 (0.03)	0.04 (0.01)
Murder of strangers	0.49 (0.06)	0.6 (0.07)	0.08 (0.02)	0.02 (0.01)
Kidnapped	0.48 (0.05)	0.33 (0.06)	0.10 (0.02)	0.00 (0.01)
Tortured	0.77 (0.05)	0.25 (0.07)	0.13 (0.03)	0.01 (0.01)
